# Cognitive and physical impairment and the risk of stroke – A prospective cohort study

**DOI:** 10.1038/s41598-020-63295-y

**Published:** 2020-04-14

**Authors:** A. Heshmatollah, U. Mutlu, P. J. Koudstaal, M. A. Ikram, M. K. Ikram

**Affiliations:** 1 Department of Epidemiology, Erasmus MC University Medical Center, Rotterdam, The Netherlands; 2 Department of Neurology, Erasmus MC University Medical Center, Rotterdam, The Netherlands

**Keywords:** Epidemiology, Neurodegeneration, Stroke, Stroke, Risk factors

## Abstract

The manifestation of cognitive and physical impairment in stroke patients before the acute event suggests accumulating subclinical vascular pathology in the brain. We investigated whether impairments in cognitive and physical functioning were associated with an increased stroke risk. Between 2002 and 2008, 8,519 stroke-free non-demented participants from the population-based Rotterdam Study underwent cognition and physical assessments including Mini-Mental State Examination, 15-word learning test, Stroop test, letter-digit substitution test, verbal fluency test, Purdue pegboard test and questionnaires on basic and instrumental activities of daily living (BADL; IADL). Principal component analysis was used to derive global cognition (G-factor). Incident stroke was assessed through continuous monitoring of medical records until 2016. Among 8,519 persons (mean age 66.0 years; 57.8% women), 489 suffered a stroke during mean follow-up of 8.7 years (SD: 2.9). Worse G-factor was associated with higher stroke risk (Hazard Ratio 1.21, 95% CI: 1.06–1.38), largely driven by unspecified stroke. Likewise, worse scores on 15-word learning test, Stroop test, Purdue pegboard test, IADL, and BADL were associated with higher risk of stroke. Thus both worse cognitive and physical functioning were associated with a higher stroke risk, in particular unspecified stroke and persons with worse memory, information processing, executive function, and motor function.

## Introduction

Stroke is a leading cause of disability and mortality worldwide and its burden is expected to rise due to aging populations^1,2^. Post-stroke cognitive and physical deficits lead to dementia in one out of three stroke survivors and impairments in activities of daily living (ADL) in over half of the patients^3,4^. Besides post-stroke manifestations, cognitive deterioration was present in about 9–14% of the patients before their stroke and patients showed a faster decline in physical independency years prior to their stroke compared to stroke-free persons^5,6^.

Cognitive and physical deterioration are thus suggested to represent early manifestations of accumulating subclinical vascular pathology in the brain prior to the acute event. Previous studies have indeed linked impairments in global cognitive and physical functioning with an increased risk of stroke^5,7,8^. Moreover, cognitive domains are indicated to be differentially affected by subclinical vascular pathology, with arteriolosclerotic damage thought to primarily affect executive domains and amyloid β being more related to memory^9^. Daily activities can also be divided into different domains including tasks that require mainly mobility, e.g. walking, and more cognitively challenging tasks, such as doing finances. However, earlier studies have generally relied on global markers of cognitive and physical functioning or used a limited number of tests that do not represent all domains^5,10–12^. Furthermore, ischemic pathology typically occurs due to arteriosclerotic and thrombotic processes, whilst the hemorrhagic component is affected by small vessel disease including amyloid β accumulation in the vessel wall^13^. Yet current literature does not differentiate between stroke subtypes.

We therefore investigated whether impairments in different domains of cognitive and physical functioning were associated with an increased risk of different stroke subtypes in community-dwelling individuals.

## Methods

### Study population

**Figure 1:**
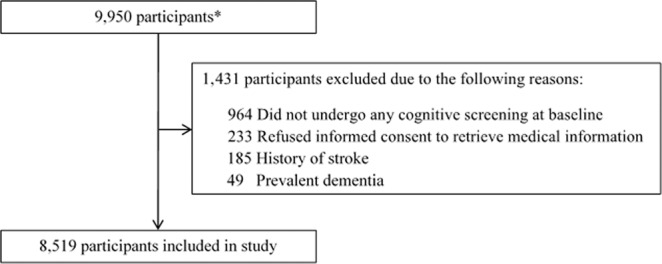
Flowchart of participants who met the inclusion/exclusion criteria. *Including participants of all subcohorts from 2002 until 2004 (fourth visit of the first subcohort, second visit of the second subcohort and first visit of the third subcohort).

This study is embedded in the Rotterdam Study, an ongoing prospective population-based cohort study in the Netherlands with the aim to investigate causes and determinants of diseases in elderly^14^. The cohort was initiated in 1990 and was expanded in 2000 and 2006, with a total of 14,926 participants aged 45 years or older. Baseline for the present study was formed by all participants from 2002 until 2008 (fourth visit of the first subcohort, second visit of the second subcohort and first visit of the third subcohort, n = 9,950), when extensive cognitive assessment was introduced. We excluded participants who did not visit the research center during this period and thus did not undergo cognitive assessment, physical examinations and blood sampling (n = 903), participants who refused informed consent for reviewing medical records for incident stroke, dementia or coronary heart disease (n = 233), and participants with prior stroke (n = 185) or prior dementia (n = 49). This resulted in a study population of 8,519 participants (Fig. 1).

The Rotterdam Study has been approved by the institutional review board (Medical Ethics Committee) of the Erasmus Medical Center and by the review board of the Netherlands Ministry of Health, Welfare and Sports. All participants provided written informed consent and all research was performed in accordance with relevant guidelines and regulations. The first author had full access to all the data in the study and takes responsibility for its integrity and the data analysis.

### Assessment of cognition

During their visit to the research center, participants underwent routine cognitive assessment, including the Mini-Mental State Examination (MMSE) and a battery of cognitive tests including 15-word learning test (immediate recall, delayed recall, and recognition), Stroop test (reading, interference, and color naming task; error-adjusted time in seconds), letter-digit substitution test, verbal fluency test (animal categories), and Purdue pegboard test (both hands simultaneously)^15^. These tests tap into different cognitive domains, namely memory (15-word learning test), information processing (Stroop reading and color naming task, letter-digit substitution test), executive function (Stroop interference task, verbal fluency test) and fine motor function (Purdue pegboard test). The distribution of all tests was transformed into a normal standardized distribution and a Z-score for every individual was calculated. We used two different measurements for global cognition: the MMSE and the G-factor. The G-factor is a standardized compound score that is calculated using the principal component analysis. For tests with multiple subtasks, only one subtask was included in order to prevent highly correlated tasks distorting the factor loadings. The tests included were verbal fluency test, 15-word learning test (only delayed recall), letter-digit substitution test, Stroop test (only color naming task) and Purdue pegboard test. The G-factor explained 52.4% of the variance in our study population.

### Assessment of physical functioning

Physical functioning was assessed with questionnaires on basic and instrumental activities of daily living (BADL and IADL). BADL was assessed with the disability index from the Stanford Health Assessment Questionnaire, which includes 20 items constituting eight components of physical activity: arising, dressing and grooming, hygiene, eating, walking, grip, reach, and activities^16^. Items were scored from 0–3 with higher scores indicating worse ability (0; no difficulty, 1; some difficulty, 2; much difficulty, 3; unable to do). Component scores were calculated as the highest scored item in that component. BADL score was determined by summing all components, obtaining a score between 0 and 24. IADL was assessed with the Lawton and Brody Instrumental Activities of Daily Living scale^17^. This scale represents activities that require more cognitive capacity, and is divided into eight components: shopping, meal preparation, laundry, medication maintenance, management of finances, housekeeping, traveling alone and using a telephone. These items were scored likewise from 0 to 3 (from no difficulty to unable to do). IADL score was obtained by summing all components, resulting in a score between 0 and 24. If participants reported that they did not perform certain activities, these items were scored as non-applicable (15.6% of the IADL variables). To prevent selective loss of data, these items were imputed by the mean of five imputations, based on age, sex, all BADL items and all other available IADL items. Finally, both BADL and IADL scores were standardized into Z-scores with higher scores indicating higher independence.

### Assessment of stroke

Stroke was defined according to the World Health Organization criteria as a syndrome of rapidly developing clinical signs of focal (or global) disturbance of cerebral function, with symptoms lasting 24 hours or longer or leading to death, with no apparent cause other than of vascular origin^18^. History of stroke at baseline was assessed during baseline interview and verified by reviewing medical records. After enrollment, participants were continuously monitored for incident stroke through automated linkage of the study database with files from general practitioners. Nursing home physicians’ and general practitioner’s files of participants who moved out of the district were checked on a regular basis as well. Additional information was obtained from hospital records. Potential strokes were reviewed by research physicians, and verified by an experienced stroke neurologist. Strokes were further classified as ischemic or hemorrhagic stroke based on neuroimaging reports or hospital discharge letters, and unspecified if these were absent. This classification corresponded with ICD-10 codes I61, I63 and I64. Participants could contribute person-years to the follow-up for a maximum of 14 years, that is, from baseline until a first-ever stroke occurred, or until death, or, if lost to follow-up, until their last health status update when they were known to be free of stroke, whichever came first, or until January 2016. Follow-up was virtually complete (95.8%).

### Assessment of dementia

The screening and follow-up for dementia has been extensively described elsewhere^19^. In short, participants were screened for dementia at baseline with the MMSE and the Geriatric Mental State Schedule (GMS). Screen-positives (MMSE < 26 or GMS > 0) underwent examination and information review using the Cambridge Examination of Mental Disorders in the Elderly. A final dementia diagnosis was decided by a consensus panel led by an experienced neurologist. Participants were also continuously monitored for incident dementia through automated linkage of the study database with files from general practitioners and the regional institute for outpatient mental healthcare.

### Other measurements

Trained research physicians visited all participants at home for filling in standardized questionnaires about health status, medication use and medical history. Body mass index (BMI) was calculated as weight (kg) divided by the square of height (m^2^). Blood pressure was measured at the right upper arm using a sphygmomanometer during two consecutive readings and the average of the two readings was used for further analysis. Blood samples were drawn to assess lipid and glucose levels, and apolipoprotein ε4 carriership. Diabetes mellitus type 2 was defined as a fasting glucose of ≥7.0 mmol/L, a non-fasting or post-load serum glucose of ≥11.1 mmol/L, or blood glucose-lowering medication use. Smoking behavior was classified as current, past or never, and alcohol consumption was calculated in gram per day. Education was divided into primary, lower/intermediate education, intermediate vocational or higher general education, and higher vocational education or university. To maximize our statistical power, missing data on covariates (except for apolipoprotein ε4 carriership) was imputed using five-fold multiple imputation, based on determinants, outcome, and covariates. The percentage of missing values in covariates ranged from 0.4% to 3.4%.

### Statistical analysis

Cox proportional–hazards models were used to estimate proportional hazard ratios of incidence stroke per unit decrease of baseline levels of cognitive and physical functioning. The proportional hazard assumption was met. Participants were censored at the time of any stroke in all initial analyses. In addition, we also performed a sub-analysis by additionally censoring for dementia. For analysis on the seven cognitive tests separately, we determined the number of independent tests using the correlation matrix of these seven tests, which resulted in 6.3 independent tests. This value was used to calculate a Bonferroni corrected p-value threshold of p = 0.0079 (0.05/6.3). We adjusted for age and sex (model I) and additionally for systolic and diastolic blood pressure, blood pressure-lowering medication, total cholesterol, high-density lipoprotein cholesterol, lipid-lowering medication use, BMI, diabetes mellitus type 2, smoking, alcohol use, level of education, and apolipoprotein ε4 carriership (model II). We furthermore estimated hazard ratios for stroke per unit decrease in cognitive and physical functioning after stratification for apolipoprotein ε4 carriership and sex. Finally, as a sensitivity analysis, we used validated cut-off points of baseline levels of MMSE, BADL and IADL and estimated the hazard ratios for stroke incidence in each group. All data analysis was performed using IBM SPSS version 21 and R 3.4.0 software.

## Results

Baseline characteristics of the study population are summarized in Table 1. Mean age of the 8,519 participants was 66.0 years (SD: 10.4), of whom 57.8% were women. During a mean follow-up of 8.7 years (SD: 2.9) a total of 489 strokes occurred, including 363 (74.2%) ischemic strokes, 62 (12.7%) hemorrhagic strokes and 64 (13.1%) unspecified strokes. Lower MMSE scores were not associated with a higher risk of any stroke (Table 2). However, lower G-factor at baseline showed a significant association with a higher risk of any stroke (per SD decrease HR 1.21, 95% CI: 1.06–1.38). Also, lower scores on both BADL and IADL were significantly associated with an increased risk of any stroke. These results did not differ across model I and II (model I not shown). After additional censoring for incident dementia, the association between G-factor and any stroke was attenuated whereas the associations between both ADL scores and any stroke remained present. Analysis on separate stroke subtypes revealed strong associations between G-factor and BADL score and risk of unspecified stroke (Table 3). BADL score was also significantly associated with the risk of ischemic stroke. After additional censoring for incident dementia the results remained stable and the association between IADL and the risk of unspecified stroke was strengthened and became significant. The analysis on separate cognitive tests showed that the 15-word learning test, all three Stroop subtasks, and the Purdue pegboard test were significantly associated with a higher risk of any stroke (Table 4). The Stroop interference task however did not survive multiple testing correction. Participants with lower scores on the Purdue pegboard test had the highest risk of stroke (per SD decrease HR 1.27, 95% CI: 1.13–1.44). Furthermore, all separate cognitive tests were significantly associated with a higher risk of unspecified stroke. Stratification for apolipoprotein ε4 carriership showed strong associations between G-factor, BADL and IADL and the risk of unspecified stroke in participants who were apolipoprotein ε4 carriers. It also revealed an interaction in the association between G-factor and risk of unspecified stroke (p-value for interaction 0.044) (Table 5). Stratification for sex showed no interaction (data not shown). Furthermore, repeating the analysis with validated cut-off points for baseline levels of MMSE, BADL and IADL showed that patients beneath these thresholds, hence those with cognitive impairment and moderate to severe impairments in daily activities, had a higher risk of stroke than those without these impairments.(Supplementary Table S1). These hazard ratios were higher than those estimated in the per SD unit decrease analysis.

**Table 1:** Baseline characteristics of the 8,519 participants.

Characteristic	Descriptive
Age, mean (SD), years	66.0 (10.4)
Women, No. (%)	4,921 (57.8)
Body mass index, mean (SD), kg/m^2^	27.7 (4.3)
Systolic blood pressure, mean (SD), mm Hg	142.7 (22.1)
Diastolic blood pressure, mean (SD), mm Hg	81.0 (11.1)
Blood pressure-lowering drugs, No. (%)	3,156 (37.0)
Diabetes mellitus type 2, No. (%)	1,068 (12.5)
Total cholesterol, mean (SD), mmol/L	5.6 (1.0)
High-density lipoprotein, mean (SD), mmol/L	1.4 (0.4)
Lipid-lowering drugs, No. (%)	1,832 (21.5)
Smoking, No. (%)	
Current	1,650 (19.4)
Former	4,291 (50.3)
Never	2,578 (30.3)
Alcohol, mean (SD), g/day	10.6 (12.8)
Education, No. (%)	
Primary	942 (11.1)
Low/intermediate	3,456 (40.6)
Intermediate	2,490 (29.2)
Higher	1,631 (19.1)
Apolipoprotein (*ApoE)* ε4 carrier*	2,302 (27.0)

No = number; SD = standard deviation. *The apolipoprotein ε4 carriership measurement was missing in 314 participants (3.7%).

**Table 2:** Associations of cognitive and physical functioning with risk of stroke, additionally censored for incident dementia.

	Any stroke	Any stroke (dementia censored)
**Cognitive functioning**		
MMSE (per point decrease)		
n/N	476/8,194	437/8,194
HR (95% CI)	1.03 (0.99–1.06)	1.01 (0.96–1.05)
G-factor (per SD decrease)		
n/N	359/6,907	331/6,907
HR (95% CI)	**1.21 (1.06–1.38)**	1.13 (0.99–1.30)
**Physical functioning**		
BADL (per SD decrease)		
n/N	455/7,312	417/7,312
HR (95% CI)	**1.18 (1.07–1.29)**	**1.20 (1.09–1.33)**
IADL (per SD decrease)		
n/N	476/8,180	437/8,180
HR (95% CI)	**1.10 (1.01–1.19)**	**1.10 (1.01–1.20)**

BADL = Basic Activities of Daily Living; CI = confidence interval; HR = hazard ratio; IADL = Instrumental Activities of Daily Living; MMSE = Mini-Mental State Examination; SD = standard deviation.

Higher scores on MMSE and G-factor indicate better cognitive function. Higher scores on ADL indicate higher independence. All analyses were censored for incident stroke (any stroke) and additionally for incident dementia.

Significant associations are displayed in bold text. Model II is shown, results did not differ across model I and II. Adjusted for age, sex, systolic and diastolic blood pressure, blood pressure-lowering medication, total cholesterol, high-density lipoprotein cholesterol, lipid-lowering medication use, BMI, diabetes mellitus type 2, smoking, alcohol use, level of education and apolipoprotein e4 carriership.

**Table 3:** Associations of cognitive and physical functioning with risk of stroke subtype, additionally censored for incident dementia.

	Ischemic stroke	Ischemic stroke (emented censored)	Hemorrhagic stroke	Hemorrhagic stroke (dementia censored)	Unspecified stroke	Unspecified stroke (dementia censored)
**Cognitive functioning**						
MMSE (per point decrease)						
n/N	352/8,194	334/8,194	61/8,194	56/8,194	63/8,194	47/8,194
HR (95% CI)	1.02 (0.98–1.07)	1.01 (0.96–1.06)	0.94 (0.81–1.08)	0.88 (0.75–1.03)	1.06 (0.98–1.15)	1.04 (0.94–1.15)
G-factor (per SD decrease)						
n/N	271/6,907	258/6,907	44/6,907	41/6,907	44/6,907	32/6,907
HR (95% CI)	1.10 (0.94–1.28)	1.07 (0.91–1.26)	1.06 (0.72–1.58)	0.93 (0.61–1.43)	**1.82 (1.39–2.38)**	**1.73 (1.24–2.42)**
**Physical functioning**						
BADL (per SD decrease)						
n/N	335/7,312	318/7,312	61/7,312	56/7,312	59/7,312	43/7,312
HR (95% CI)	**1.13 (1.01–1.26)**	**1.15 (1.02–1.29)**	1.08 (0.82–1.42)	1.08 (0.81–1.44)	**1.36 (1.09–1.69)**	**1.59 (1.24–2.03)**
IADL (per SD decrease)						
n/N	353/8,180	335/8,180	61/8,180	56/8,180	62/8,180	46/8,180
HR (95% CI)	1.04 (0.94–1.15)	1.03 (0.93–1.14)	1.11 (0.88–1.41)	1.14 (0.89–1.45)	1.20 (1.00–1.44)	**1.33 (1.08–1.63)**

BADL = Basic Activities of Daily Living; CI = confidence interval; HR = hazard ratio; IADL = Instrumental Activities of Daily Living; MMSE = Mini-Mental State Examination; SD = standard deviation. Higher scores on MMSE and G-factor indicate better cognitive function. Higher scores on ADL indicate higher independence. All analyses were censored for incident stroke (any stroke) and additionally for incident dementia. Significant associations are displayed in bold text. Model II is shown, results did not differ across model I and II. Adjusted for age, sex, systolic and diastolic blood pressure, blood pressure-lowering medication, total cholesterol, high-density lipoprotein cholesterol, lipid-lowering medication use, BMI, diabetes mellitus type 2, smoking, alcohol use, level of education and apolipoprotein e4 carriership.

**Table 4:** Associations of separate cognitive tests with risk of stroke subtype.

Cognitive test (per SD decrease)	Any stroke	Ischemic stroke	Hemorrhagic stroke	Unspecified stroke
**Memory**				
15-Word learning test				
n/N	415/7,397	308/7,397	52/7,397	55/7,397
HR (95% CI)	**1.17 (1.04–1.32)**	1.13 (0.99–1.31)	0.90 (0.63–1.29)	**1.64 (1.21–2.23)**
**Information processing**				
Stroop reading task				
n/N	405/7,408	296/7,408	52/7,408	57/7,408
HR (95% CI)	**1.13 (1.04–1.23)**	1.05 (0.93–1.18)	0.94 (0.69–1.28)	**1.37 (1.22–1.55)**
Stroop color naming task				
n/N	405/7,408	296/7,408	52/7,408	57/7,408
HR (95% CI)	**1.10 (1.05–1.16)**	1.03 (0.91–1.16)	1.08 (0.87–1.35)	**1.17 (1.10–1.24)**
Letter-digit substitution test*				
n/N	438/7,889	328/7,889	58/7,889	52/7,889
HR (95% CI)	1.08 (0.96–1.21)	1.02 (0.89–1.17)	0.93 (0.68–1.29)	**1.72 (1.19–2.48)**
**Executive function**				
Stroop interference task				
n/N	402/7,394	296/7,394	52/7,394	54/7,394
HR (95% CI)	1.10 (1.02–1.19)^†^	1.03 (0.93–1.15)	0.98 (0.74–1.28)	**1.26 (1.14–1.41)**
Verbal fluency test				
n/N	422/7,660	313/7,660	53/7,660	56/7,660
HR (95% CI)	1.09 (0.97–1.22)	1.04 (0.91–1.18)	0.88 (0.64–1.21)	**1.81 (1.30–2.52)**
**Motor function**				
Purdue pegboard test				
n/N	422/7,725	317/7,725	54/7,725	51/7,725
HR (95% CI)	**1.27 (1.13–1.44)**	**1.17 (1.02–1.35)**	1.34 (0.95–1.88)	**1.79 (1.28–2.48)**

CI = confidence interval; HR = hazard ratio; SD = standard deviation. Higher scores on cognitive tests indicate better cognitive function. All analyses were censored for incident stroke (any stroke). Significant associations after correction for multiple testing are displayed in bold text. Model II is shown, results did not differ across model I and II. Adjusted for age, sex, systolic and diastolic blood pressure, blood pressure-lowering medication, total cholesterol, high-density lipoprotein cholesterol, lipid-lowering medication use, BMI, diabetes mellitus type 2, smoking, alcohol use, level of education and apolipoprotein e4 carriership.

*Some studies include the letter-digit substitution test in the executive function domain.

^†^Associations with p < 0.05 that did not survive multiple testing correction.

**Table 5:** Associations of cognitive and physical functioning with risk of stroke subtype, stratified for apolipoprotein ε4 carriership.

	Ischemic stroke		Hemorrhagic stroke		Unspecified stroke	
	ApoE4 carrier	ApoE4 non-carrier	ApoE4 carrier	ApoE4 non-carrier	ApoE4 carrier	ApoE4 non-carrier
**Cognitive functioning**						
MMSE (per point decrease)						
n/N	83/2,298	269/5,896	22/2,298	39/5,896	14/2,298	49/5,896
HR (95% CI)	1.01 (0.91–1.12)	1.02 (0.97–1.08)	0.99 (0.80–1.23)	0.90 (0.75–1.09)	1.08 (0.87–1.34)	1.07 (0.98–1.16)
G-factor (per SD decrease)						
n/N	67/1,829	204/4,938	15/1,829	29/4,938	10/1,829	34/4,938
HR (95% CI)	1.01 (0.73–1.41)	1.12 (0.94–1.34)	0.76 (0.36–1.60)	1.23 (0.78–1.93)	**5.58 (2.31–13.52)**	**1.65 (1.23–2.21)**
**Physical functioning**						
BADL (per SD decrease)						
n/N	80/2,024	255/5,288	22/2,024	39/5,288	12/2,024	47/5,288
HR (95% CI)	1.02 (0.79–1.31)	**1.17 (1.03–1.33)**	1.27 (0.81–1.99)	0.99 (0.69–1.40)	**2.01 (1.13–3.58)**	**1.29 (1.01–1.66)**
IADL (per SD decrease)						
n/N	83/2,293	270/5,887	22/2,293	39/5,887	14/2,293	16/5,887
HR (95% CI)	1.02 (0.82–1.26)	1.05 (0.94–1.18)	1.14 (0.78–1.67)	1.09 (0.80–1.47)	**1.52 (1.03–2.24)**	1.08 (0.86–1.35)

ApoE4 = Apolipoprotein ε4; BADL = Basic Activities of Daily Living; CI = confidence interval; HR = hazard ratio; IADL = Instrumental Activities of Daily Living; SD = standard deviation.

Higher scores on MMSE and G-factor indicate better cognitive function. Higher scores on ADL indicate higher independence. All analyses were censored for incident stroke (any stroke). Significant associations are displayed in bold text. Adjusted for age, sex, systolic and diastolic blood pressure, blood pressure-lowering medication, total cholesterol, high-density lipoprotein cholesterol, lipid-lowering medication use, BMI, diabetes mellitus type 2, smoking, alcohol use, and level of education.

## Discussion

In this prospective population-based cohort study, we found that worse cognitive and physical functioning were associated with a higher risk of stroke, in particular for the risk of unspecified stroke. With respect to specific cognitive domains, these associations were present in persons with poorer performance on memory, information processing, executive function, and motor function.

Previous studies showed that a lower score on MMSE or a comparable routine cognitive screening test was associated with an increased risk of stroke^10–12,20,21^. In our study, the G-factor showed a significant association with the risk of stroke and not the MMSE. An explanation could be that the MMSE, although commonly used in practice, is relatively insensitive for mild cognitive impairment and focuses mostly on orientation and memory^22^ whereas the G-factor is thought to be a more comprehensive summary of a person’s cognitive functioning.

Furthermore, we found similar associations with the risk of stroke across different cognitive domains that are thought to be linked to arteriolosclerotic and amyloid β accumulation, suggesting that both pathways underlie the pathophysiology of stroke. In the ARIC study, cognition was assessed through the Delayed Word Recall test, the Digit Symbol Subtest and the Word Fluency test, which underlie memory, psychomotor performance and linguistic impairment, respectively. In an initial analysis no association was found between cognitive impairment and risk of stroke^23^. However, a subsequent analysis from the ARIC Study, that included all cardiovascular events (stroke, myocardial infarctions or coronary heart disease related death) did find a significant association with lower scores on the cognitive tests, suggesting that the first study may have been underpowered^24^. The Framingham study used a battery of cognitive tests and had a study population similar to the present study^25^. Deficit in executive function was associated with an increased risk of stroke. The other cognitive tests suggested an association with stroke, but did not reach statistical significance.

Two large prospective studies on basic physical functioning, measured with questionnaires including walking, dressing and bathing, also found an association with physical dependence and risk of stroke^5,26^. However, these findings could not be replicated in another prospective study including 9,451 persons^20^. The BADL score and the Purdue pegboard test, both representing motor function, showed strong associations with the risk of stroke in our study. Impaired motor function may be a result of structural brain changes, as disability has previously been linked to an increased all-cause mortality risk^27,28^.

The IADL score, which includes more cognitively challenging tasks, was also associated with the risk of stroke. A previous study used general markers on the intellectual activity of persons, such as reading newspapers, and found a similar association with the risk of stroke^7^. The association with IADL was mostly attenuated after additional censoring for dementia, strengthening the suggestion that a low IADL score is partially a result of cognitive impairment.

Focusing on the risk of different stroke subtypes, the effect sizes were the largest for unspecified strokes. As mentioned before, we classified a stroke as unspecified when there was no neuroimaging conducted or available. In general, these unspecified stroke cases were those in which the general practitioners or the nursing home physicians did not refer the patient to the hospital, due to severe paresis or because participants were frailer with poor overall health due to extensive co-morbidities for which an elaborate evaluation in the hospital was not desirable. This may explain why we found the strongest associations in this subgroup.

An underlying pathophysiological mechanism for our findings could be that cognitive and physical impairment share a common cause with stroke due to accumulating cerebral vascular pathology^9,29,30^. Pre-existent vascular risk factors result in cerebral small vessel disease such as lacunar infarcts and white matter hyperintensities, which can lead to an increased risk of stroke and also to cognitive and physical impairment^9,29^. Cerebral small vessel disease and subsequently arterial stiffness are considered potential causes of cerebral hypoperfusion, which is associated with accelerated cognitive decline and increased risk of dementia^30^. Another explanation of our results could be through neurodegeneration. We showed that apolipoprotein ε4 carriers were at a much higher risk of unspecified stroke than non-carriers, implying that β-amyloid accumulation could underlie this association. This could again be through arterial stiffening and impaired vasodilatation that occur as a result of the β-amyloid deposition^31^. Additional censoring for incident dementia attenuated the association of G-factor with incident stroke risk, again suggesting an underlying neurodegenerative component. In contrast, the ADL scores and Purdue pegboard test results were not attenuated implicating that neurodegenerative processes seem to play a more limited role in their association with stroke.

Several limitations of this study need to be discussed: First, as not all participants performed all sub-tasks listed on the questionnaires on IADL, a total of 15.6% of these variables were missing. However, by imputing these data using the other covariates including the BADL scores, we prevented selective loss of data. Second, as is the case in other population-based studies, we had a relatively large number of unspecified stroke cases in which we could not determine the specific subtype due to lacking neuroimaging^32^. Strengths of our study include: The prospective design with an average follow-up of 8.7 years with nearly complete follow-up and the availability of extensive cognitive tests and questionnaires on both basic and instrumental ADL. The latter resulted in a reliable estimate of cognition and physical functioning per individual.

## Conclusions

We found that both worse cognitive and physical functioning were associated with a higher risk of stroke in the general population. Poorer performance on specific cognitive tests that reflect memory, information processing, executive function and motor function showed a similar association. Cognitive and physical testing could be a potential marker of pre-existing cerebrovascular pathology.

## Supplementary information

Supplementary Tables.

## Data Availability

Rotterdam Study data can be made available to interested researchers upon request. Requests can be directed to data manager Frank J.A. van Rooij (f.vanrooij@erasmusmc.nl). We are unable to place data in a public repository due to legal and ethical restraints. Sharing of individual participant data was not included in the informed consent of the study, and there is potential risk of revealing participants’ identities as it is not possible to completely anonymize the data. This is of particular concern given the sensitive personal nature of much of the data collected as part of the Rotterdam Study.
