# Supplementary material for: Cognitive and physical impairment and the risk of stroke – A prospective cohort study
Source: Sci Rep. 2020 Apr 14;10:6274. doi: 10.1038/s41598-020-63295-y (PMC7156475; doi:10.1038/s41598-020-63295-y)
Supplement: Supplementary file 1 — Supplementary Tables. [file 41598_2020_63295_MOESM1_ESM.pdf]

Cognitive and physical impairment and the risk of stroke – A prospective cohort study

A. Heshmatollah, MD,[a,b] U. Mutlu, MD PhD,[a] P.J. Koudstaal, MD PhD,[b] M.A. Ikram, MD PhD,[a] M.K. Ikram, MD PhD.[a,b]

[a] Department of Epidemiology, Erasmus MC University Medical Center, Rotterdam, The Netherlands.

[b] Department of Neurology, Erasmus MC University Medical Center, Rotterdam, The Netherlands.

Supplementary Tables

Table S1. Association of cognitive and physical functioning with risk of stroke.

	**n/N**	**Any stroke**<br>HR (95% CI)
**MMSE**		
No cognitive impairment (24-30)	445/7,871	1.00 [reference]
Cognitive impairment (0-24)	31/323	1.21 (0.83 – 1.76)
**BADL**		
None to mild impairment (0-8)	378/6652	1.00 [reference]
Moderate to severe impairment (9-24)	77/660	**1.40 (1.07 – 1.84)**
**IADL**		
None to mild impairment (0-8)	427/7795	1.00 [reference]
Moderate to severe impairment (9-24)	49/385	1.28 (0.94 – 1.75)

BADL=Basic Activities of Daily Living; CI=confidence interval; HR=hazard ratio; IADL=Instrumental Activities of Daily Living, MMSE=Mini-Mental State Examination.

Significant associations are displayed in bold text. Model II is shown, results did not differ across model I and II. Adjusted for age, sex, systolic and diastolic blood pressure, blood pressure-lowering medication, total cholesterol, high-density lipoprotein cholesterol, lipid-lowering medication use, BMI, diabetes mellitus type 2, smoking, alcohol use, level of education and apolipoprotein e4 carriership.
